# Association between developmental milestones and age of schizophrenia onset: Results from the Northern Finland Birth Cohort 1966

**DOI:** 10.1016/j.schres.2019.02.013

**Published:** 2019-06

**Authors:** Jan Stochl, Anjalene Whittier, Adam P. Wagner, Juha Veijola, Erika Jääskeläinen, Jouko Miettunen, Golam M. Khandaker, Peter B. Jones

**Affiliations:** a Department of Psychiatry, University of Cambridge, Cambridge, UK; b National Institute for Health Research Collaboration for Leadership in Applied Health Research and Care East of England, Cambridge, UK; c Department of Kinanthropology, Charles University, Prague, Czech Republic; d Norwich Medical School, University of East Anglia, Norwich, UK; e Center for Life Course Health Research, University of Oulu, Oulu, Finland; f Medical Research Center Oulu, Oulu University Hospital and University of Oulu, Oulu, Finland; g Department of Psychiatry, Oulu University Hospital, Oulu, Finland; h Department of Psychiatry, Research Unit of Clinical Neuroscience, University of Oulu, Oulu, Finland; i Cambridgeshire and Peterborough NHS Foundation Trust, Cambridge, UK

**Keywords:** Developmental milestones, Schizophrenia, Non-affective psychoses, Age at onset

## Abstract

We investigated relationships between early developmental milestones, schizophrenia incidence and variability in its age at onset. We hypothesized that the period of risk for schizophrenia would be longer for those with later development. The Northern Finland Birth Cohort 1966 was followed until 47 years of age, and those members diagnosed with schizophrenia or any other non-affective psychoses identified. Latent profile analysis was used to classify people into homogenous classes with respect to developmental milestones, and subsequently survival analysis explored relationship between classes and age of schizophrenia onset. Results suggest that 4-classes (early, regular, late, and extra late developers) can be identified, but due to few cases in one class (n = 93, <0.01% of 10,501), only 3 classes (early, regular, late) could be meaningfully compared. Schizophrenia incidence until 47 years of age differed systematically between classes: late developers had the highest cumulative incidence (2.39%); regular were intermediate (1.25%); and early developers had the lowest incidence (0.99%). However, age at onset and its variability was similar across classes, suggesting that our hypothesis of a wider ‘window’ for schizophrenia onset in late developers was not supported.

## 1 Introduction

Considerable scientific research implicates impaired neurodevelopment in the aetiology of schizophrenia and other psychotic disorders (Murray and Lewis, 1988; Weinberger, 1987). Evidence from population-based longitudinal studies linking obstetric complications (Cannon et al., 2002b), delayed motor development (Isohanni et al., 2001; Jones et al., 1994), lower premorbid IQ (Cannon et al., 2002a; Cannon et al., 2000; David et al., 1997; Davidson et al., 1999; Jones et al., 1994; Reichenberg et al., 2005; Zammit et al., 2004), language and social development (Bearden et al., 2000; Crow et al., 1995) supports a neurodevelopmental facet to the origin of schizophrenia. A meta-analysis of population-based studies reported that there is a linear association between premorbid IQ and risk of schizophrenia (Khandaker et al., 2011), which has been replicated in large-scale national epidemiological samples recently (Kappelmann et al., 2018; Kendler et al., 2015; Khandaker et al., 2018). Furthermore, lower premorbid IQ is associated with an earlier age of illness onset for schizophrenia (Khandaker et al., 2011).

Age at onset appears at least partially to impact the outcomes of schizophrenia. Earlier development of schizophrenia is predictive of severe cognitive decline (Rajji et al., 2009), higher social and intellectual impairment (Johnstone et al., 1989), and more negative symptoms, hospitalizations, and relapses (Immonen et al., 2017). Psychotic disorders typically emerge in early adulthood, with a median age at onset and the majority of manifestations occurring during patients' twenties (Kessler et al., 2007; Thorup et al., 2007). Still, a number of cases occur beyond this window of peak risk, but are typically studied only cross-sectionally or retrospectively. Few studies have had the opportunity to examine data collected across such a wide range of life stages but one, the Northern Finland Birth Cohort 1966 (NFBC 1966; Rantakallio, 1969) has benefitted from over half a century of prospective data collection. Studies such as these have used large samples followed up at regular intervals across the span of multiple decades, allowing researchers to investigate risk factors across the lifespan (Jääskeläinen et al., 2015).

Indeed, seminal groundwork regarding motor milestones and the development of psychosis has arisen from the NFBC 1966 (Isohanni et al., 2001) and several other cohorts (Jones et al., 1994). It is well-established in the literature that delayed childhood motor development is a risk factor for adult psychosis. A recent meta-analysis of five large-scale birth cohort studies found that later ages of first walking unsupported, standing unsupported, and sitting unsupported were significantly associated with adult onset schizophrenia (Filatova et al., 2017a). Two notable publications included in this meta-analysis examined data from the NFBC 1966 including 152 cases of schizophrenia and 10,131 controls followed prospectively from birth until the age of 46 (Isohanni et al., 2001; Keskinen et al., 2015). A more recent study in the same cohort found that delayed motor milestones were associated with an increased risk of schizotypy (Filatova et al., 2017b). Other evidence from the Dunedin (Cannon et al., 2002a), 1961–1969 Helsinki (Clarke et al., 2011), 1959–1961 Copenhagen (Sørensen et al., 2010), and 1946 British (Jones et al., 1994) birth cohorts linking late developmental milestones to an increased risk of developing schizophrenia has consistently established that these associations exist across cultures and timeframes.

While studies have examined the association between the timing of milestone attainment and diagnosis of schizophrenia or schizotypy, the post-pubertal and early adult period during which the schizophrenia syndrome is first manifest has had less attention. This is surprising given that the rapid deceleration in risk after the mid-twenties is as dramatic as the acceleration during the teenage years (Thorup et al., 2007). If later milestones in childhood represent delayed neuronal maturation during the early years that may, through common mechanisms, increase risk for schizophrenia during the post-pubertal epoch, this aberrant process may, similarly, lead to a protracted period of risk. We hypothesized that, alongside higher incidence, there would be larger variance in the age at which late developers are first diagnosed with psychosis.

## 2 Materials and methods

### 2.1 The Northern Finland Birth Cohort 1966

The Northern Finland Birth Cohort 1966 (NFBC 1966) is composed of 12,058 live births in Oulu and Lapland born in 1966 (96% of births in the region during this period), followed prospectively from pregnancy onwards (Rantakallio, 1969). Data have been obtained on an ongoing basis from questionnaires, clinical examinations, and health and governmental records. In this study we analysed data from 10,501 individuals (5370 (51.1%) males and 5131 (49.9%) females) with *any* available information on developmental milestones. Within this subset, mean birth height and weight were 50.2 cm (median = 50, sd = 2.4) and 3490 g (median = 3500, sd = 530) respectively. 491 (4.8%) individuals were born early (<36 weeks) and 122 (1.1%) suffered from perinatal brain damage.

A considerable amount of data was missing on milestones (see Table 1 for percentage of missing data and Supplementary Table 1 for its most common patterns) such as being capable of standing up, grabbing objects or touching the thumb with the index finger. However, there were no statistically or clinically significant differences between individuals with complete and missing developmental milestones data with respect to birth weight, gestation age, or brain damage. The only statistically significant difference was found with birth height (Holm corrected p-value = 0.03) but the actual difference was clinically marginal (complete cases: mean = 50.37 cm, median = 50 cm, sd = 2.21 cm; cases with missing data: mean = 50.21 cm, median = 50 cm, sd = 2.43 cm). Descriptive statistics of developmental milestones are provided in Table 1.

Until the year 2013 (when the cohort became 47 years old), schizophrenia was diagnosed in 148 individuals (mean onset age = 27.5 yrs, sd = 7.5 yrs), and non-schizophrenic psychoses in 235 individuals (mean onset age = 35.0 yrs, sd = 8.4 yrs).

**Table 1:** Descriptive statistics for developmental milestones in months, ordered bv average age of achievement.

Milestone	N	% of missing data^a^	Mean	sd	Median	Min	Max
Making sounds	6010	42.77	1.40	0.57	1	1	6
Able to hold head up	5890	43.91	2.14	0.74	2	1	7
Grab object	5634	46.35	3.25	0.70	3	1	9
Turning from back to tummy	6348	39.55	4.38	1.10	4	1	11
Sitting without support	10,234	2.54	6.58	1.77	6	0	14
Touch thumb with index finger	5625	46.43	7.25	1.15	7	2	14
Capable to stand up	3504	66.63	7.43	1.33	7	1	13
Walking with support	9865	6.06	9.20	1.44	9	4	19
Standing without support	8771	16.47	10.43	1.36	10	6	24
Walking without support	8280	21.15	11.51	1.56	11	7	28

a Percentage of missing data from entire sample (n = 10,501).

### 2.2 Measures

#### 2.2.1 Developmental milestones

The following developmental milestones were available in the NFBC 1966 and utilised in the analysis: making sounds; being able to hold head up when arms lifted; making a grip on an object (grab object); turning from back to tummy; sitting without support; touching thumb with index finger (like a tweezer); capable of standing up (lift themselves up); walking with support; standing without support; and walking without support. For each of the milestones, the month in which it is achieved is recorded for each individual. This data was gathered during monthly visits to the Finnish child welfare clinics (mean number of 10 visits during first year of life) where nurses and doctors interviewed (using unstructured interview) the parents and systematically observed the children (Keskinen et al., 2015). This information was merged with parental responses to a questionnaire on motor development gathered at one year of age (Isohanni et al., 2001; Rantakallio et al., 1985).

#### 2.2.2 Diagnosis of psychotic disorder

Diagnoses of psychotic disorders were collected from all available nationwide registers, including inpatient and outpatient hospital visits (Care Register for Health Care) and registers for Social Insurance Institute (Filatova et al., 2017c; Keskinen et al., 2015). The diagnoses were categorized following the conventions of the ICD-8, ICD-9, and ICD-10 for the years in which they were utilised. Diagnoses were collected up until December 2013. Further information on how these classifications were defined are available from previous studies (Keskinen et al., 2015).

We established two psychosis outcome groups. A schizophrenia group comprised all those individuals with diagnosis of schizophrenia (ICD-10 code F20). A non-schizophrenia psychosis group included individuals with any other non-affective psychosis (ICD-10 codes: F21-F25, F28, F29, F302, F312, F315, F323, F333).

### 2.3 Analysis

Latent profile analysis (LPA), with developmental milestones used as indicators, was used to identify a number of homogeneous, distinct classes of individuals based on their developmental milestones. The entire sample (n = 10,501) was included as LPA can utilise individuals with partially missing data. Maximum likelihood estimation with 50 random starts was used to minimize the risk of finding local maxima. We tested models with up to seven classes. The choice between models – equivalently, the number of classes – was based on: Akaike information Criteria (AIC; Akaike, 1974); Bayesian Information Criteria (BIC; Schwarz, 1978); BIC's sample size adjusted variant (SABIC); classification entropy; and the distribution of individuals across classes. Individuals were allocated into classes based on the largest probability (maximum a posteriori, MAP). LPA was conducted using MPlus 8 (Muthén and Muthén, 1998–2017). Levene's test and Kruskal-Wallis tests were used to test whether variances and means of schizophrenia onset age are equal across classes. Finally, survival analysis and Cox regression were carried out in R (R Core Team, 2017) using the *survival* package (Therneau, 2015) to assess differences in incidence between classes.

## 3 Results

### 3.1 Latent profile analysis: population sub-groups of infant development

Table 2 shows the fit of the LPA models, differing by the number of classes fitted in each model.

**Table 2:** Information criterions (lower values preferred) and entropy (values closest to 1 preferred) for the LPA models differing in the number of classes.

LPA model	AIC	BIC	SABIC	Entropy
2-class	212,551	212,776	212,678	0.681
3-class	209,119	209,424	209,291	0.735
4-class	207,026	207,411	207,242	0.770
5-class	205,881	206,346	206,142	0.746
6-class	204,829	205,373	205,135	0.696
7-class	a	a	a	a

AIC = Akaike information criterion, BIC = Bayesian information criterion, SABIC = Sample size adjusted BIC.

a Convergence issues.

For determining the number of classes, the information criterions were inconclusive: both AIC and BIC kept reducing as the number of classes fitted increased. However, with seven or more classes, there were convergence issues, preventing further reliable comparisons. Classification entropy suggested a four-class model for well-separated classes.

**Fig. 1:**
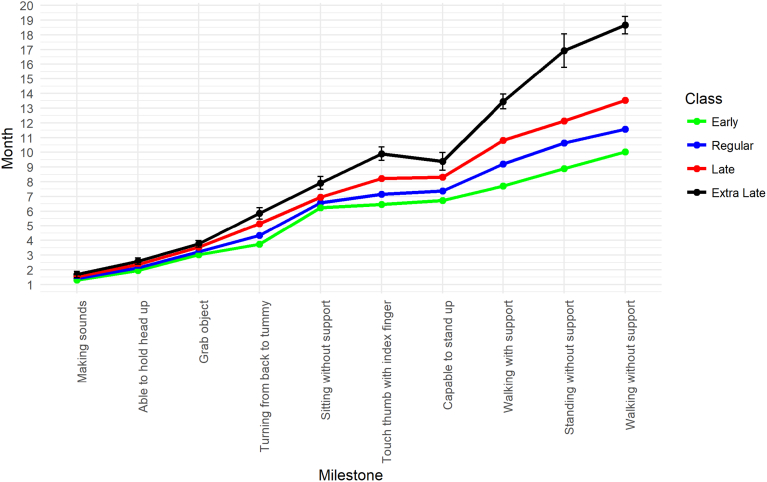
Means and 95% confidence intervals for developmental milestones by class (four-class solution).

Table 3 shows the distribution of individuals across models with two to four classes. Class labels were based on comparisons between class means for each milestone. Fig. 1 shows the mean age of achieving each milestone by class for the four-class models.

**Table 3:** The distribution of the 10,501 individuals with developmental milestones data across classes.^a^

LPA model	Early	Regular	Late	Extra late
2-class	–	6137	4364	–
3-class	3351	6195	955	–
4-class	2416	5911	2081	93

a For the 5- and 6-class models it became difficult to differentiate between classes prohibiting meaningful interpretation. These solutions are therefore not presented.

The extra late class consisted of 93 individuals, of whom only two developed schizophrenia (at 26 and 37). The class was therefore unsuitable for meaningful assessment of variability of age of schizophrenia onset and survival analysis. Thus, we merged the late and extra late classes into a single class labelled as ‘late’.

### 3.2 Size of the developmental window for schizophrenia

**Fig. 2:**
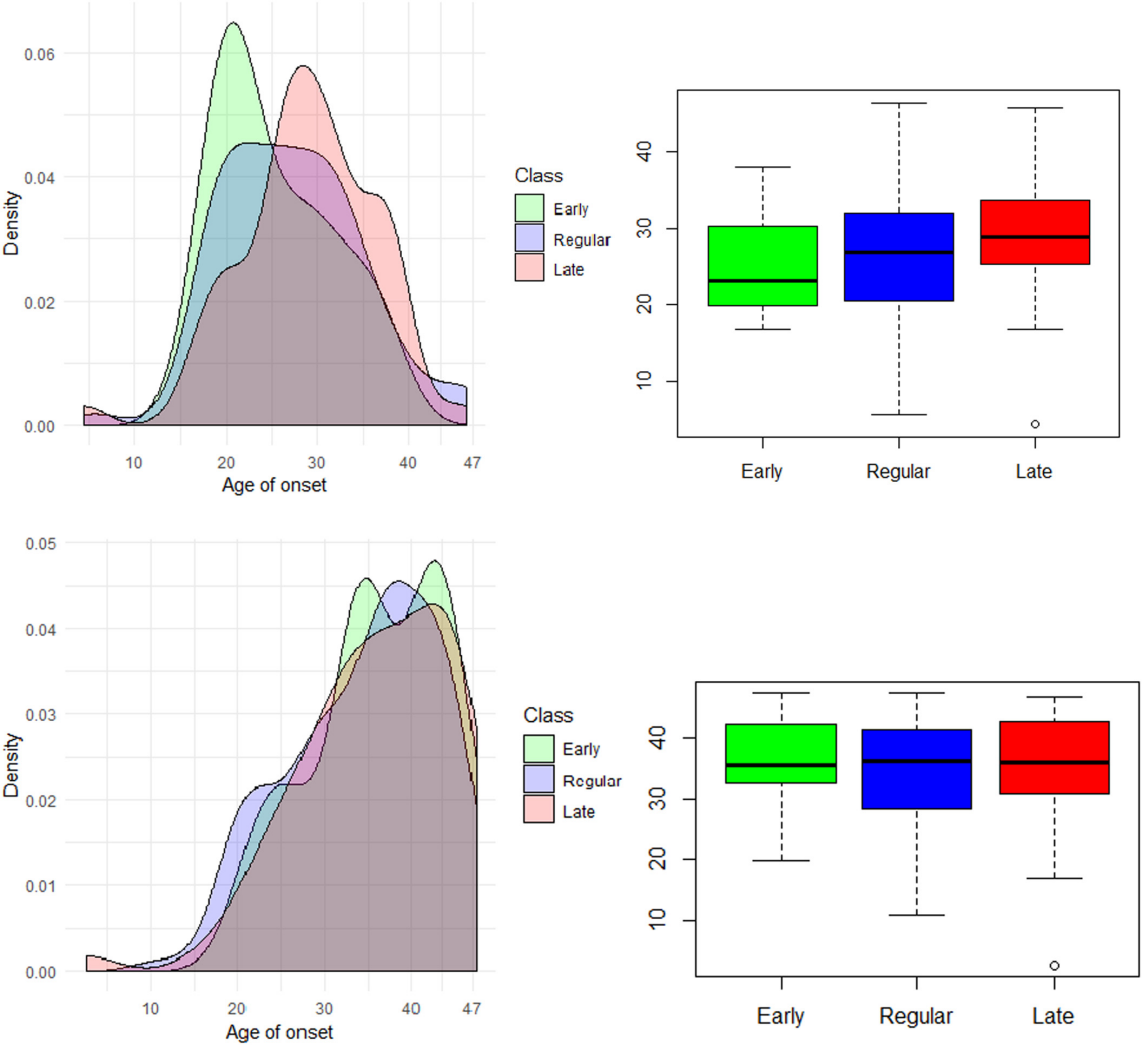
Distribution of age at onset of schizophrenia (top) and non-schizophrenic psychoses (bottom) across classes; density plot (left) and boxplot (right).

We hypothesized that individuals with delayed development may have a wider ‘window’ during which they remain at risk of schizophrenia onset compared with individuals with typical development. Analytically, this means testing whether variances of schizophrenia onset age are different between classes. Levene's test of homogeneity of variances suggested no difference in variance of schizophrenia onset (p = 0.660). We have plotted class densities and box plots of onset age to illustrate this (Fig. 2).

The order of distribution peaks suggested that delayed early development might be related to delayed onset of schizophrenia. However, a Kruskal-Wallis test showed no statistically significant differences in age at onset (p = 0.075).

### 3.3 Survival analysis: age at schizophrenia onset

**Fig. 3:**
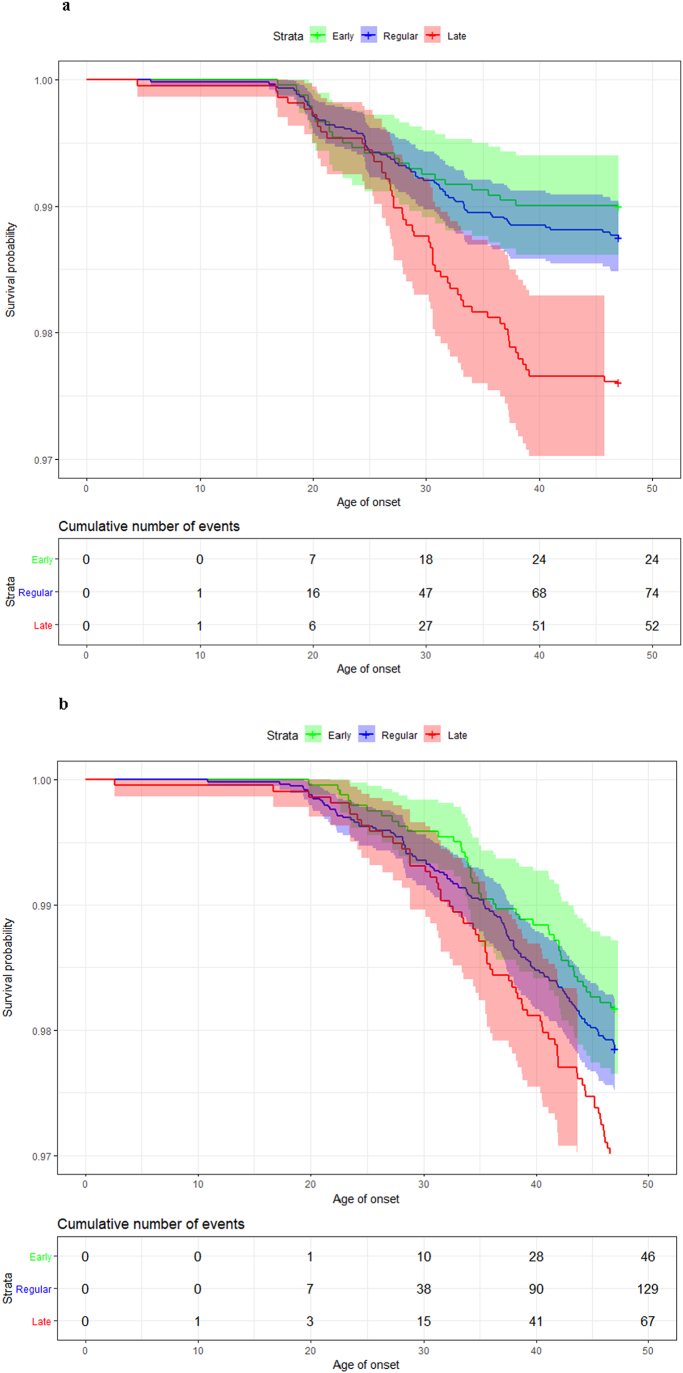
a: Survival function curves (with confidence intervals) and table of cumulative incidence of schizophrenia across classes. b: Survival function curves (with confidence intervals) and table of cumulative incidence of non-schizophrenic psychoses across classes.

Another way to compare the length of time over which members of different classes remain at risk is to use Cox regression and compare survival curves, as shown in Fig. 3a. Generally, the hazard for schizophrenia onset decreases with earlier development. The hazard ratio (HR) between regular and early developmental classes was 0.79 (the risk of schizophrenia onset is decreased by 21% in individuals reaching milestones early), but this was not statistically significant (p = 0.324). Compared with regular developers, late developers have almost twice the risk (HR = 1.92, p < 0.001). Finally, the hazard for schizophrenia onset is 2.42 times larger for late developers compared with early developers (HR = 2.42, p < 0.001). The log-rank test gives statistically significant (p < 0.001) differences between survival curves. Visual inspection of survival curves, such as comparing curve shapes and noting the x-axis range over which changes are occurring, also supports that the variance of schizophrenia onset age is similar across classes.

### 3.4 Comparison with non-schizophrenia psychoses

The bottom panel of Fig. 2 and Fig. 3b show density comparison and boxplots, and survival curves for people with non-schizophrenic psychoses. These show that onset age and its variance are similar across classes (Kruskal-Wallis p = 0.369; Levene's test p = 0.756), and survival curves across classes are more similar than for schizophrenia although the difference remains statistically significant (log-rank p = 0.010). The risk of having non-schizophrenic psychoses is decreased by 15% (HR = 0.85) for the early development class compared to regular developers (although this difference is statistically non-significant, p = 0.372). The risk is increased by 44% (HR = 1.44, p = 0.015) for late developers compared to the regular developmental class and by 68% (HR = 1.68, p = 0.007) compared to the class of early developers.

## 4 Discussion

This study investigates the relationship between early development and age of schizophrenia onset. Typically, schizophrenia is diagnosed between 20 and 35 years of age: its incidence peaks between 10 and 25 years for men, and between 25 and 35 for women (Rajji et al., 2009). We hypothesized that this onset ‘window’ may be wider for individuals with delayed early development. Results do not support this hypothesis.

To address this hypothesis, we allocated individuals into four classes, denoted as early, regular, late and extra late. However, the extra late class consisted of few individuals (<0.01%, 93/10,501) of whom only two developed schizophrenia. Such small classes, consisting of <1% of the sample size, are not usually considered adequate for comparison (Nylund et al., 2007). Thus, we merged extra late and late classes into a single class.

Levene's test of homogeneity of schizophrenia onset age and comparison of onset age densities revealed no differences in variability of onset age between classes. There is a trend in mean onset age: later infant development is associated with later schizophrenia onset. However, differences between groups were non-significant.

Survival analysis showed statistically significant differences in schizophrenia incidence between classes. Individuals with delayed development have larger hazards than both the early (HR = 2.42) and regular (HR = 1.92) classes. This pattern continues: individuals in the early development class had a lower, though not statistically significant, hazard of schizophrenia onset than compared to those in the regular class (HR = 0.79). Finally, visual inspection of survival curves confirmed that onset age variability is similar across classes. The pattern is similar for incidence of non-schizophrenic psychoses, though the differences in risks across groups are smaller.

Overall, these findings are largely consistent with past studies on developmental delay and schizophrenia risk (Filatova et al., 2017a; Keskinen et al., 2015), and add further weight to an association between motor developmental classes to risk of clinical diagnosis over time. While we did not find the same association for non-schizophrenic psychoses, this could indicate that something unique to schizophrenia is driving this relationship. More work is needed to identify the precise nature of these delays.

In our sample, the pattern of association with developmental milestones is similar for both schizophrenia and other psychotic disorders (i.e., delayed milestones increase risk), but the effect of developmental delay is more pronounced for schizophrenia. This may indicate a dose-response pattern such that strength of association increases when a stricter outcome definition is used. It could also reflect true underlying biological distinctions between schizophrenia and other psychotic disorders. However, previous population-based longitudinal studies of premorbid IQ suggest that premorbid IQ deficit is similar between schizophrenia and other non-affective psychosis (Kendler et al., 2015; Khandaker et al., 2018). This would argue against different mechanisms. Previous studies of developmental milestones support this view. In the NFBC 1966 birth cohort, ages at learning to stand, walk and become potty-trained were each related to subsequent incidence of both schizophrenia and other psychoses, but not with non-psychotic disorders (Isohanni et al., 2001). In a Danish cohort, individuals who later developed psychiatric disorders other than schizophrenia reached most developmental milestones earlier than those who developed schizophrenia, but later than the controls (Sørensen et al., 2010).

It is possible that IQ is a mediator between developmental milestones and risk of schizophrenia. Reaching developmental milestones at later ages in childhood is correlated with having a lower IQ across the lifespan (Murray et al., 2007). A modest deficit exists in premorbid IQ scores for people with schizophrenia and can be measured reliably even in childhood (Jones et al., 1994; Khandaker et al., 2011; Khandaker et al., 2018; Woodberry et al., 2008). Furthermore, a linear relationship has been found between premorbid IQ scores and schizophrenia risk, with lower scores at higher risk compared to normal IQs, and higher IQs at lower risk compared to normal IQs (Zammit et al., 2004). Delayed milestones and premorbid IQ deficits could also be risk indicators for schizophrenia. For instance, a previous study from the NFBC 1966 showed that infant motor development and adult cognitive performance are underpinned by frontal cortico-cerebellar connectivity in healthy individuals. Disruption of this anatomical system may underlie both the early developmental and cognitive abnormalities in schizophrenia (Ridler et al., 2006). Further work from the same cohort supports that in schizophrenia mild infant motor developmental delay and adult cognitive deficits (at least in some domains) are age dependent manifestations of the same underlying neural process (Murray et al., 2006). Furthermore, in babies who, as adults, suffered schizophrenia or any psychosis, those who learned to stand latest were also more likely to perform poorly at school in both motor and theoretical domains at age 16 when compared with earlier learners (Isohanni et al., 2004).

Deterioration in academic functioning prior to onset has been identified (Allen et al., 2005), and neuroinflammatory patterns similar to those found in neurodegenerative disorders have been revealed (Pasternak et al., 2012). Our study did not examine this pathway, but future researchers might be interested in this question or, alternatively, might examine whether IQ mediates the relationship between delayed milestones and schizophrenia. Studies of prenatal/early childhood infection, developmental milestones are also required. Exposure to infection in childhood is associated with IQ deficit at age 18 years, which partly mediates association between childhood infection and adult non-affective psychosis (Khandaker et al., 2018).

In any case, cognition is clearly implicated. There has been longitudinal work implicating various forms and manifestations of cognitive impairment in schizophrenia, with some even arguing these deficits are the defining feature of the illness – perhaps even above and beyond the psychotic elements (Elvevag and Goldberg, 2000; Kahn and Keefe, 2013). While certainly not all children who develop at later ages can be said to be cognitively impaired, our study has demonstrated that there is an association present within these cases that begins in the earliest months of life. If these findings are shown to be consistent, the possibility that indicators of this may be visible early in life could help future researchers develop early interventions or monitor strategies for children at high genetic risk of developing schizophrenia.

Limitations include the inconclusive results of class enumeration in LPA. The goodness of fit statistics (BIC and SABIC), which are considered relevant indices for this purpose (Nylund et al., 2007), suggest adding additional classes until convergence issues. This may suggest class separation is not clear, or that the distribution of motor development is not a mixture of distributions. Indeed, in models with five or more classes, some classes were difficult to differentiate and interpret. The proposed classes in this study clearly suggested that infants are early, regular, late or extra late consistently across all milestones. We were not able to find a class with children who were, for example, late in earlier milestones (e.g. those achieved usually before 6 months) but then accelerated their development in later milestones.

In conclusion, schizophrenia incidence (but not other types of psychoses) was different between classes (decreasing from late developers, through to regular and early ones). However, regardless of type of psychosis, age at onset variability was similar across classes, suggesting that our hypothesis of a wider ‘window’ for schizophrenia onset in late motor developers is not supported, and that the determinants of early motor development are distinct from those that influence psychosis syndrome onset age.

The following is the supplementary data related to this article.

Supplementary Table 1 The most frequent missing data patterns (1 = observed, 0 = missing). Supplementary Table 1

## Funding body agreements and policies

NFBC 1966 received financial support from University of Oulu Grant no. 24000692, Oulu University Hospital Grant no. 24301140, ERDF European Regional Development Fund Grant no. 539/2010 A31592. AP Wagner, J Stochl, and PB Jones received support from the NIHR Collaboration for Leadership in Applied Health Research and Care (CLAHRC) East of England (EoE) at the Cambridgeshire and Peterborough NHS Foundation Trust. A Whittier is supported by the Gates Cambridge Trust. J Miettunen is supported by the Academy of Finland (grant nr. 268336). Dr. Khandaker acknowledges funding support from the Wellcome Trust (Intermediate Clinical Fellowship; grant no: 201486/Z/16/Z), MQ: Transforming Mental Health (grant no: MQDS17/40), and the UK Medical Research Council (grant no: MC_PC_17213). E Jääskeläinen received grants from the Academy of Finland (grant number 278 286), the Sigrid Jusélius Foundation, the Brain & Behavior Research Foundation.

The views expressed are those of the authors and not necessarily those of the NHS, the NIHR or the Department of Health and Social Care.

## Contributors

JS undertook the statistical analysis and with AW wrote the first draft of the manuscript. PBJ designed the study. APW reviewed the statistical analyses and GK reviewed the theoretical backgrounds and managed literature searches. JV, EJ, and JM provided data. All authors contributed to and have approved the final manuscript.

## Conflict of interest

All authors declare that they have no conflicts of interest.
