# Supplementary material for: Association between developmental milestones and age of schizophrenia onset: Results from the Northern Finland Birth Cohort 1966
Source: Schizophr Res. 2019 Jun;208:228–34. doi: 10.1016/j.schres.2019.02.013 (PMC6551363; doi:10.1016/j.schres.2019.02.013)
Supplement: Supplementary Table 1 — The most frequent missing data patterns (1 = observed, 0 = missing). [file mmc1.docx]

**Supplementary Table 1: The most frequent missing data patterns (1=observed, 0=missing).**

| **Frequency** | **Making sounds** | **Able to hold**  **head up** | **Grab object** | **Turning from back to tummy** | **Sitting**  **without support** | **Touch thumb with index finger** | **Capable to**  **stand up** | **Walking**  **with support** | **Standing**  **without support** | **Walking**  **without support** |
| --- | --- | --- | --- | --- | --- | --- | --- | --- | --- | --- |
| **1873** | 1 | 1 | 1 | 0 | 0 | 1 | 0 | 0 | 0 | 0 |
| **1057** | 1 | 1 | 1 | 0 | 1 | 1 | 1 | 1 | 1 | 1 |
| **632** | 0 | 1 | 1 | 0 | 0 | 1 | 0 | 0 | 0 | 0 |
| **507** | 0 | 0 | 1 | 0 | 0 | 1 | 0 | 0 | 0 | 0 |
| **330** | 1 | 1 | 1 | 0 | 0 | 1 | 1 | 1 | 1 | 1 |
| **177** | 1 | 1 | 1 | 0 | 1 | 1 | 1 | 0 | 1 | 1 |
| **155** | 1 | 1 | 1 | 1 | 0 | 1 | 1 | 1 | 1 | 1 |
| **141** | 1 | 1 | 1 | 0 | 1 | 1 | 1 | 0 | 0 | 0 |
| **134** | 0 | 0 | 0 | 0 | 0 | 1 | 0 | 0 | 0 | 0 |
| **106** | 0 | 1 | 1 | 1 | 1 | 1 | 1 | 1 | 1 | 1 |
